# Pickering Particles Prepared from Food Waste

**DOI:** 10.3390/ma9090791

**Published:** 2016-09-21

**Authors:** Joanne Gould, Guillermo Garcia-Garcia, Bettina Wolf

**Affiliations:** 1 Division of Food Sciences, School of Biosciences, The University of Nottingham, Sutton Bonington Campus, Loughborough LE12 5RD, UK; bettina.wolf@nottingham.ac.uk; 2 Centre for SMART, Wolfson School of Mechanical and Manufacturing Engineering, Loughborough University, Loughborough LE11 3TU, UK; G.Garcia-Garcia@lboro.ac.uk

**Keywords:** pickering emulsions, particles, lignin, food emulsions

## Abstract

In this paper, we demonstrate the functionality and functionalisation of waste particles as an emulsifier for oil-in-water (o/w) and water-in-oil (w/o) emulsions. Ground coffee waste was chosen as a candidate waste material due to its naturally high content of lignin, a chemical component imparting emulsifying ability. The waste coffee particles readily stabilised o/w emulsions and following hydrothermal treatment adapted from the bioenergy field they also stabilised w/o emulsions. The hydrothermal treatment relocated the lignin component of the cell walls within the coffee particles onto the particle surface thereby increasing the surface hydrophobicity of the particles as demonstrated by an emulsion assay. Emulsion droplet sizes were comparable to those found in processed foods in the case of hydrophilic waste coffee particles stabilizing o/w emulsions. These emulsions were stable against coalescence for at least 12 weeks, flocculated but stable against coalescence in shear and stable to pasteurisation conditions (10 min at 80 °C). Emulsion droplet size was also insensitive to pH of the aqueous phase during preparation (pH 3–pH 9). Stable against coalescence, the water droplets in w/o emulsions prepared with hydrothermally treated waste coffee particles were considerably larger and microscopic examination showed evidence of arrested coalescence indicative of particle jamming at the surface of the emulsion droplets. Refinement of the hydrothermal treatment and broadening out to other lignin-rich plant or plant based food waste material are promising routes to bring closer the development of commercially relevant lignin based food Pickering particles applicable to emulsion based processed foods ranging from fat continuous spreads and fillings to salad dressings.

## 1. Introduction

Pickering particles are solid particles capable of stabilising an emulsion by the adsorption of solid particles to the oil/water interface. The application of Pickering particles has attracted significant research interest in recent years as unlike molecular emulsifiers, which constantly adsorb and desorb from the interface promoting emulsion droplet coalescence, Pickering particles are considered to be irreversibly adsorbed. This is because the free energy needed for spontaneous desorption of particles from the interface is extremely large compared to that of thermal energy. For example, for desorption of a particle of radius 10 nm adsorbed at a toluene-water interface with a contact angle of 90° the energy required is 2750 KT [1]. The particle is therefore considered to be permanently adsorbed as the high desorption energy means a high energy input is needed to disrupt the particle layers to allow droplet coalescence to occur. This holds true for all particle stabilised emulsions even for small nanoparticles (r ≈ 5–10 nm) as long as the contact angle of the particle is not too close to 0° or 180° [2].

The properties of particle stabilised emulsions (droplet size, flocculation, viscosity) are majorly influenced by the properties of the particles and emulsion phases controlling the arrangement of the particles at the interface. Particle wettability is a key determinant of whether an oil-in-water (o/w) or a water-in-oil (w/o) emulsion is obtained, commonly characterised by the contact angle at the interface measured through the water phase. Particles classed as hydrophilic adopt a contact angle of less than 90° at a planar air/water or oil/water interface, i.e., these are preferentially wetted by water. Conversely, particles forming contact angles of greater than 90° are hydrophobic and are wetted by the oil phase to a greater extent [3,4]. During emulsion formation, the interface of a droplet will curve to ensure the larger area of the particle surface remains on the external side, such that hydrophilic particles will give rise to o/w emulsions and hydrophobic particles to w/o emulsions [3,4].

Utilisation of Pickering particles in emulsion based foods and other consumer good products, e.g., creams and lotions, offers several advantages such as replacement of artificial surfactants, prolonged shelf life, and stabilisation of complex structures such as multiple emulsions. However, the inclusion of these particles in food products is hampered through the lack of interfacially active food particles. Hydrophobic OSA modified starches [5], flavonoids [6], chitin nanocrystals extracted from crab shells [7], fat particles such as hardened rapeseed oil particles [8], protein based particles [9], protein microgels [10], egg yolk granules [11], and colloidal cellulose based fibers [12] have all been shown to have interfacial functionality although most often chemical modification is required before use. We have, on the other hand, recently demonstrated that particles from the shell and nib of the Theobroma Cacao pod act as Pickering particles. O/W emulsions readily formed during high shear mixing processes showed no evidence of a change in emulsion droplet size over 100 days of storage or the presence of an oil layer after storage for two years, indicating the formation of a highly stable microstructure [13,14]. These particles are not only food grade but also natural as there is no requirement for chemical modification. Further investigations of these natural Pickering particles indicated that the emulsifying ability of the particles was enhanced by the presence of lignin.

Lignin is the second most abundant natural polymer after cellulose, characterised by its highly branched heterogeneous structure built from aromatic residues. It is widely considered to be a hydrophobic molecule, however it has also been shown to have hydrophilic, hydrophobic, and amphiphilic character depending on botanical origin and extraction methods [15,16]. Kraft lignins [17,18], lignosulfonates [18], lignin obtained from enzymatic hydrolysis [19] and lignin microparticles [20,21] have been shown to stabilise o/w emulsions. Several methods have been used to create lignin microparticles; one such method uses aqueous ethanol to extract lignin from shrub willow and an anti-solvent precipitation protocol to prepare the microparticles. The emulsifying ability of the lignin microparticles was then assessed in a soybean oil-in-water system with the result being the formation of stable o/w emulsions with no significant change in droplet size over a storage period of five months [20].

To the best of our knowledge there have yet to be published reports on the preparation of lignin based Pickering particles for the application in w/o emulsions warranting microstructure stability. We hypothesise that a lignin rich particulate material can be suitably processed to show functionality in this system, a functionality that we were not able to impart to cocoa particles. Their lignin content of between 4% and 9% (wt/wt) [22] is either too low or the modification methods explored in this research are not suitable. Here we selected ground coffee waste with a reported lignin content of between 20% and 27% (wt/wt) [23]. It is in plentiful supply with UK coffee shops and households producing more than 500,000 tonnes [24] and 60,379 tonnes [25] of this type of waste per year respectively. However, we foresee the waste produced during the manufacture of instant coffee, termed spent coffee grounds, also to be a suitable waste stream. Application of this technology to spent coffee grounds provides an even larger commercial potential as from the 2.5 million tonnes of coffee products manufactured in Europe in 2013, 326,320 tonnes correspond to instant coffee, with a monetary value close to €3 billion [26]. This large scale production generates over 300,000 tonnes of dry spent coffee grounds every year [27].

In order to prepare these Pickering particles we have utilised a hydrothermal treatment common to the bioenergy industry with the aim of relocating the lignin to the surface of the waste coffee particles. In doing so, we expect to increase the hydrophobicity of the particle allowing stabilisation of w/o emulsion in addition to the untreated particles stabilizing o/w emulsions. The hydrothermal treatment is carried out in water at high temperatures and pressures which causes the cell wall to be disrupted and the formation of spherical droplets on the surface of the material which are understood to be largely composed of lignin [28]. Although, lignin is notoriously complex to characterize [29] multiple techniques such as FT-IR, NMR, antibody labelling, TEM, and cytochemical staining have been used to investigate the composition of the droplets, all of which determined the droplets to be composed of lignin [30,31]. The formation of droplets occurs because, at temperatures above the melting point of lignin, typically between 100 °C and 170 °C [32], lignin fluidizes, coalesces, and has the ability to move through the cell wall matrix. Once at the surface of the sample material, the hydrophobic lignin minimizes contact with the hydrophilic solvent by forming droplets which solidify once the temperature has been brought down sufficiently [32,33]. However, some authors do conclude that, during steam explosion and dilute acid treatments. Hemicellulose and lignin degradation products combine to form lignin like droplets termed pseudo-lignin [34]. Pseudo-lignin is also considered to be hydrophobic like lignin [31,35].

In this paper, we demonstrate that particles prepared from ground coffee waste show promise to be successfully applied as a versatile Pickering emulsifier through hydrothermal processing. To the best of our knowledge, this is the first report of a natural non-fat based Pickering particle suitable for application as an emulsifier of w/o food emulsions. Data presented include size, hydrophobicity, and emulsifying ability for untreated and treated ground waste coffee particles. For the sake of brevity, ground coffee waste particles are in the following referred to as coffee particles or simply particles.

## 2. Results and Discussion

### 2.1. Properties of Prepared Coffee Pickering Particles

#### 2.1.1. Surface Morphology

**Figure 1:**
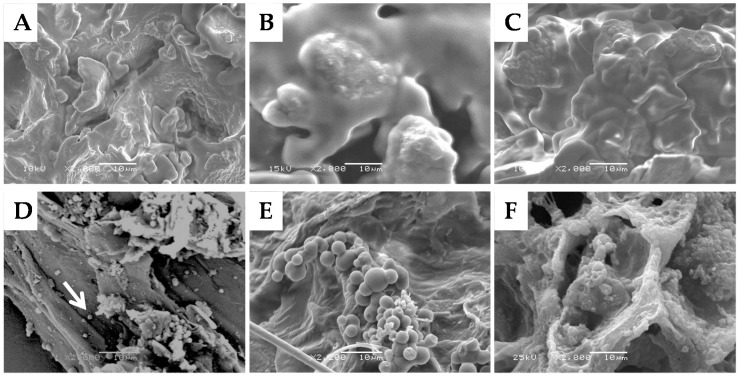
SEM images of (**A**) dried coffee particle; (**B**) dried and ball milled coffee particle and (**C**–**F**) following hydrothermal treatment at (**C**) 150 °C; (**D**) 200 °C; (**E**) 250 °C; and (**F**) 275 °C for 1 h. Scale bar represents 10 μm.

The surface morphology of a coffee particle following drying after collection (Figure 1A), that was then milled (B) and additionally submitted to hydrothermal treatment at various temperatures (Figure 1C–F) as assessed using SEM is depicted in Figure 1. The untreated coffee particle shown in Figure 1A is characterised by an irregular surface with folded rounded features. Ball milling of these particles had little effect on the surface morphology of the particles except a slight smoothing of the surface as shown in Figure 1B.

Treating the coffee particles hydrothermally at temperatures between 150 °C and 275 °C for 1 h caused the formation of droplets on the particles’ surface, which was a result of the melting and relocation of the lignin component, as discussed in the introduction. By relocating the lignin in this manner, we predict the surface hydrophobicity of the coffee particles will increase enabling the stabilisation of w/o emulsions. The effect of temperature was therefore investigated to optimize the formation of these droplets on the surface, as it has previously been reported that the density of coalesced lignin located on the cell structure of hydrothermally treated sugarcane bagasse increased with temperature [36].

Figure 1D–F demonstrate that the droplets on the surface of the coffee particles appear in clusters. Clustering around and within specific structural features such as pits, cell corners, and delamination layers has previously been reported after hydrothermal treatment of corn stem rind and explained by the porosity of these areas [30]. The pores act as extrusion channels for the melted lignin. If lignin droplets were to be found more evenly distributed across the particle surface, as has been reported for hydrothermally treated sugarcane bagasse, this would be the sign of a porous ultrastructure which may be generated in situ during hydrothermal processing due to the removal and hydrolysis of lignin and hemicellulose, respectively [36].

The amount of droplets formed on the particle surface as judged by the SEM images depended on the temperature of the hydrothermal treatment (Figure 1C–F). While the SEM image 1(C) acquired after treatment at 150 °C featured no droplets, Figure 1D indicates that hydrothermal treatment at 200 °C led to the formation of a small number of small droplets with diameters of around 1 μm. Evidenced in Figure 1E, more droplets and varying in size between 2.5 μm and 5 μm formed following treatment at 250 °C. Some droplets appear to have fused together. Also recognisable are flattened edges where a droplet may have been in close contact with another droplet that was subsequently pulled off during preparation of the sample for imaging. Similar droplet features were found on the surface of corn stover rind following hydrothermal and acid treatment [30]. Figure 1F then demonstrates that further increase in temperature is detrimental to the occurrence of surface adsorbed lignin droplets. This observation suggests that there is an optimal processing temperature to impart surface hydrophobicity to coffee particles should this indeed be the functionality of what is assumed to be mostly redistributed lignin. Therefore, the sample treated at 250 °C for 1 h was selected for further investigations.

#### 2.1.2. Lignin Content

The concentration of lignin in the particles prepared from ground coffee waste was quantified spectrophotometrically following acetyl bromide and dioxane extraction. Lignin content was 17.9% ± 1.2% in milled particles and 29.9% ± 1.2% in hydrothermally treated milled particles (250 °C for 1 h). Lignin was not created nor lost during the hydrothermal process, instead the increase in lignin content is an effect of sample mass loss due to the hydrolysis of the indigenous polysaccharides xylan and hemicellulose during hydrothermal treatment [37,38]. Such an increase has previously been reported for wheat straw following steam explosion treatment [39].

#### 2.1.3. Particle Size

The particle size of the coffee Pickering particles is shown in Table 1 as the volume weighted mean diameter, *d*_4,3_, and as a measure of the fine fraction the diameter below which 10% of the particles are found, *d*_10,3_. Ball milling decreased the values of both characteristic diameters. The hydrothermal treatment appeared to slightly increase in the mean diameter which would be the result of particle aggregation during the processing. Hydrothermal treatment did not affect the size of the fine fraction which is worth noting since our previous research on the emulsifying ability of cocoa particles has shown that the size of the fine fraction dictated the diameter of the o/w emulsions processed with these particles [13]. For a new Pickering particle system as under investigation here one would base any expectation on emulsion droplet size on the mean particle diameter. The linear relationship between emulsion droplet diameter and particle diameter stabilising the interface [40] suggests that the milled and hydrothermally treated particles would stabilise smaller emulsion droplets than the unmilled coffee particles.

#### 2.1.4. Particle Hydrophobicity

The hydrophobicity of the Pickering particles was evaluated using an emulsion assay, as measuring the contact angle, the material property commonly chosen to characterise the hydrophobicity of Pickering particles—of particles with irregular shapes—can introduce significant errors, depending on the method of assessment. Instead, the emulsion composition and processing protocol described in 3.3 was designed to give an insight into the hydrophobicity of the particles, based on the type of emulsion formed.

Unmilled and milled coffee particles stabilised o/w emulsions regardless of the oil phase composition (polarity) which was confirmed with the drop test where all emulsions dispersed in water rather than in a sample of the oil phase of the emulsion. The stabilisation of o/w emulsions by these Pickering particles indicates their hydrophilic nature, as even with the most polar oil phase (100% IPM) o/w emulsions were formed.

**Figure 2:**
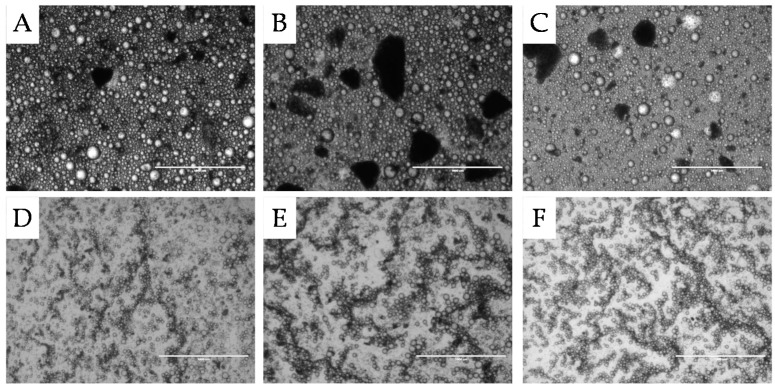
Light micrographs of o/w emulsions with 46% oil and stabilised with 8% unmilled or milled coffee particles with different oil phase polarity acquired after one day of storage at 25 °C. Images are as follows; unmilled coffee particle with an oil phase of (**A**) 100% dodecane (least polar); (**B**) 50% dodecane and 50% IPM (intermediate polarity) and (**C**) 100% IPM (most polar) and milled coffee particles (**D**) 100% dodecane; (**E**) 50% dodecane and 50% IPM and (**F**) 100% IPM. Scale bar represents 1000 μm.

Figure 2 shows the microstructures of a selection of the o/w emulsions stabilised by unmilled (Figure 2A–C) and milled coffee particles (Figure 2D–F). The micrographs show that the Pickering emulsions have a flocculated microstructure and the milling of the particles enabled the stabilisation of smaller droplets, in accordance with the smaller particle size of the milled sample reported in Table 1. Oil droplets stabilised by unmilled coffee particles showed a broad size distribution which makes differentiating the effect of oil polarity on emulsification efficacy difficult. In contrast, the size of emulsion droplets stabilised by milled coffee particles was affected by the oil phase polarity, with larger droplets stabilised when the oil phase consisted of equal quantities of IPM and dodecane as evident in Figure 2E. In absence of experimental evidence, we suggest that the altered interfacial properties or viscosity properties of the oil phase may be the reason for the formation of the larger droplets.

In contrast, emulsions processed in the presence of hydrothermally treated coffee particles formed w/o emulsions regardless of the polarity of the oil phase, again confirmed by the result of the drop test where all emulsions dispersed in the oil phase. This result demonstrates that hydrothermal treatment increased the hydrophobicity of the particles most likely due to the relocation of lignin to the particle surface.

**Figure 3:**
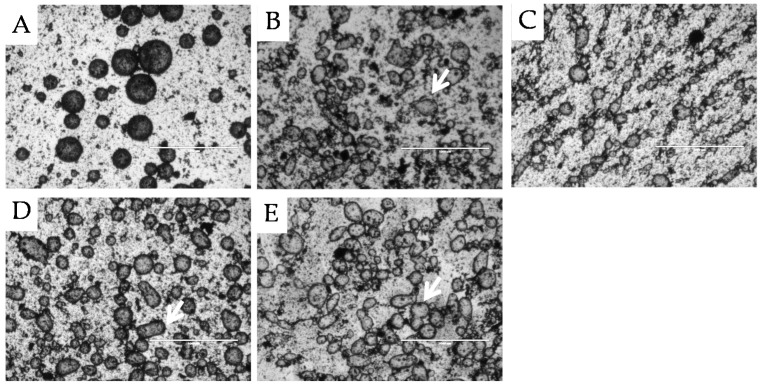
Light micrographs of w/o emulsions with 46% water and stabilised with 8% hydrothermally treated coffee particles with differing oil phase composition acquired after one day of storage at 25 °C. Images are of w/o emulsions with oil phases of (**A**) 100% dodecane; (**B**) 75% dodecane; (**C**) 50% dodecane; (**D**) 25% dodecane; and (**E**) 100% IPM. Scale bar represents 1000 μm. The arrows indicate the presence of arrested coalescence.

Figure 3 shows the microstructures of the different w/o emulsions (varying oil phase polarity) stabilised by milled and hydrothermally treated coffee particles. Comparison of Figure 3 to Figure 2 reveals obvious microstructure differences. The droplet size in the w/o emulsions was considerably larger than the droplet size in the o/w emulsions featuring droplets with diameter of between 100 μm and 300 μm. Another difference is the occurrence of irregular shaped water droplets, pointed out by the arrows in Figure 3B–E, as an intermediate stage of coalescence—termed arrested coalescence—in which droplets retain the shape of the original droplets to some extent. Complete coalescence is halted when the interface is jammed with particles preventing further reduction in interfacial area to a spherical droplet. This phenomenon is therefore strongly dependent on the level of droplet surface coverage with particles [41]. A high degree of droplet surface coverage with particles (Ø = 0.9) creates a closely packed jammed interfacial layer preventing total coalescence. In the emulsions shown in Figure 3, the droplets are described as in state of arrested coalescence. At lower surface coverage and if the combined particle covered surface area of two droplets exceeds the interfacial area that would form by complete coalescence arrested coalescence will occur. Based on experimental data, it was deducted that a combined intermediate particle surface coverage of the two droplets of 1.43 < Ø_1_ + Ø_2_ < 1.81 is required to prevent total coalescence, leaving droplets in an arrested state of coalescence [41]. The presence of irregular shaped water droplets in the case of hydrothermally treated coffee Pickering particles therefore indicates an intermediate level of water droplet surface coverage with these coffee particles.

This intermediate surface coverage may be the result of the heterogeneous distribution of lignin droplets on the particle surface evidenced in Figure 1. These overall hydrophobic Pickering particles are characterised by inhomogeneous surface chemistry, and adsorption onto the water droplet surface appears to be possible only for the more hydrophilic or lignin-poor surface domains. Particle adsorption therefore maybe characterised by large parts of the particles residing in the water phase resulting in a lower surface coverage and causing the larger size of the stabilised droplets compared to the o/w emulsions with untreated particles of a most likely relatively homogenous surface chemistry. Optimisation of the hydrothermal treatment may allow a more homogenous or control over surface chemistry/hydrophobicity allowing an increase in particle adsorption to the interface and therefore an increased surface coverage by particles, preventing arrested coalescence of emulsion droplets.

### 2.2. Application of Coffee Pickering Particles in Food Emulsions

The processability of o/w emulsions stabilised with milled waste coffee Pickering particles was evaluated to see whether coffee Pickering particles could be successfully incorporated into manufactured food products. The processability and storage stability of these Pickering emulsions was evaluated in a food emulsion formulation consisting of 46% sunflower oil and by subjecting the emulsions to shearing and heating as well as acidic and alkaline conditions. W/O emulsions were not characterised in terms of processability as the large size of the water droplets stabilised by hydrothermally treated waste coffee particles are not currently desirable in food products due to their predictable instability towards shear and mixing processes as well as potentially imparting a rough mouthfeel as the water droplets may be sensed as large solid particles due to the arrested nature of the interface.

#### 2.2.1. Microstructure Stability

**Figure 4:**
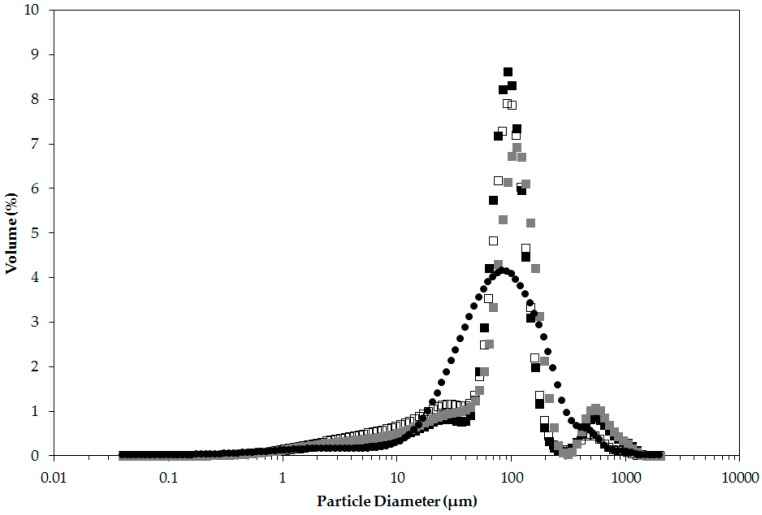
Emulsion droplet diameter volume size distribution of o/w emulsions stabilised with 8% milled waste coffee Pickering particles and sunflower oil as the oil phase (46%) acquired after 1 day (■), 6 weeks (□), and 12 weeks (■) of storage at 25 °C, is presented alongside the size distribution of the particles (●) Data presented is the mean distribution of three independent emulsion samples.

**Figure 5:**
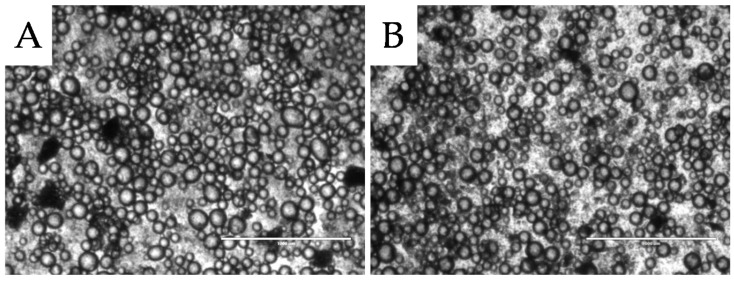
Light micrographs of o/w emulsions stabilised with 8% milled waste coffee Pickering particles and sunflower oil as the oil phase (46%) before heating (**A**); and after heating (**B**) the emulsion to 80 °C. Scale bar represents 1000 μm.

Figure 4 shows the size distribution of o/w emulsions stabilised by milled waste coffee particles measured after 1 day, 6 weeks, and 12 weeks of storage at 25 °C, alongside the size distribution of an aqueous dispersion of the milled waste coffee particles. The emulsion had a bimodal distribution with sharp peaks at 100 μm and 500 μm. It is also evident that there was a significant overlap between the size distribution of the emulsion droplets and the particles that stabilise the emulsion droplets. Based on the microscopy evidence shown in Figure 5A the peak at 100 µm can be assigned to the emulsion droplets. Due to conclusions from previous literature [2], we expect the particles that stabilise the emulsion droplets to be in the order of one magnitude smaller in diameter which would correspond to the distribution of particles below 10 µm. Particle sizes around 500 µm identify particle aggregates that can be noted in Figure 5A or individual very large particles having a large impact on particle size distribution due to weighting by volume. It is also worth noting that the difference between the emulsion and dispersion distributions between 10 µm and 30 µm is indicative of the presence of unadsorbed suspended particles in the emulsion.

As shown in Figure 4, the peak at 100 μm remained constant over the studied storage period of 12 weeks and there was no significant change in the mean diameter over storage as can be seen in Figure 7. There were minor changes in the volume fraction of the larger particles (peak around 500 µm) which we expect to be due to sampling of the large particle aggregates or individual large particles.

#### 2.2.2. Temperature Stability

To ensure products are safe for consumption high temperature processing steps such as pasteurisation, sterilisation, and cooking are often used in food manufacturing. It was therefore important to investigate the influence of heating and holding the emulsion at 80 °C for 10 min on the emulsion microstructure. Figure 5A,B presents the microstructure before and after heating, respectively. There is little difference in the degree of flocculation and droplet size evident, which was reflected in the results of the particle size analysis on these two samples (data not presented). The heat stability of the coffee particle Pickering system indicates that these emulsions could be utilised in thermally processed foods.

#### 2.2.3. Shear Stability

**Figure 6:**
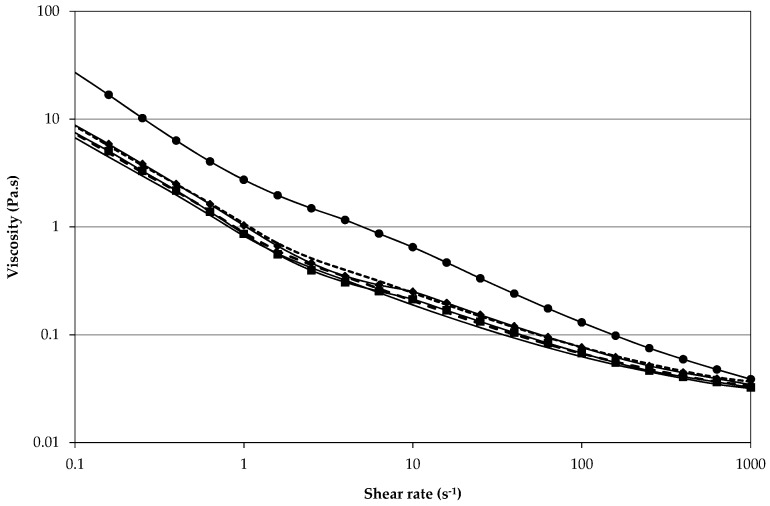
Shear stability was evaluated by applying a shear rate increase to 1000 s^−1^ and decrease to 0 s^−1^, the protocol was repeated a total of three times. Black symbols indicate shear increase steps (1st●, 2nd♦, and 3rd■) and straight lines represent shear decrease steps (1st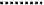, 2nd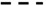, and 3rd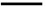).

In addition to storage and temperature stability, the microstructure of the coffee particle stabilised emulsions showed good shear stability as shown in Figure 6. The viscosity data were acquired by increasing and then decreasing the shear rate which was repeated three times in total. The emulsion is clearly shear thinning and the slight shoulder between 1 s^−1^ and 10 s^−1^ indicates that some slip occurred. Nevertheless, following the first attainment of the highest shear rate, the viscosity data overlapped at each shear rate and the viscosity recorded at the highest shear rate was constant independent of the step in the measurement sequence. This behaviour is highly indicative of the equilibration of the emulsion’s superstructure during the first shear rate increase in response to the shear rate applied, thus viscosity subsequently probed at a shear rate lower or equal to this maximum shear rate remained unchanged. There was no evidence of droplet break up caused by the shearing protocol as the mean size, characterised by the *d*_4,3_, of the emulsion droplets was not significantly (*p* < 0.05) different in emulsions before and after shearing (data not shown). Typically, it is flocculation of emulsion droplets and in this case potentially also of aggregates of non-adsorbed coffee particles that are broken up during the first shear rate increase in such up and down shear rate protocols. Indeed, the emulsion as shown in Figure 5A appears slightly flocculated.

#### 2.2.4. pH Stability of Milled Waste Coffee Pickering Emulsions

**Figure 7:**
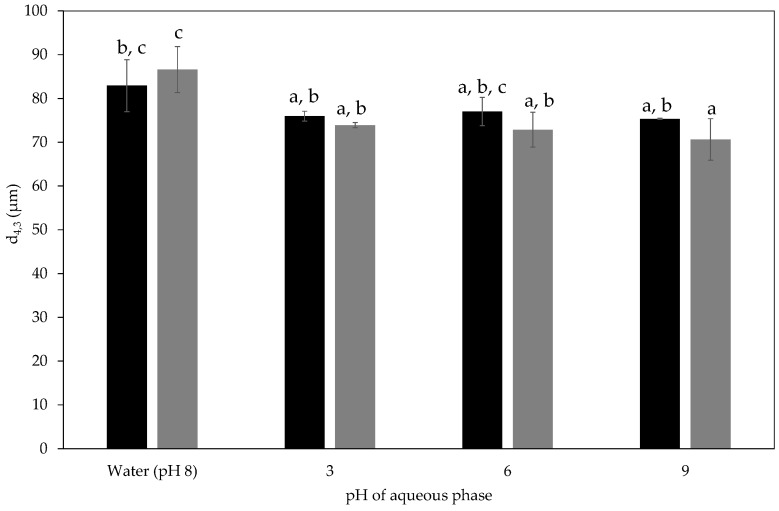
Volume mean emulsion droplet diameter acquired one day (■) and four weeks (■) after emulsification. The emulsions were formed with aqueous phases adjusted to pH 3, 6, 9. The droplet size of emulsions formed with pure water (pH 8) as the aqueous phase was included this data was acquired after one day (■) and six weeks (■). Emulsion formulation contained 8% milled waste coffee Pickering particles and 46% sunflower oil. The presence of different letters (a,b,c) represent a significant difference between samples (*p* < 0.05).

Application in manufactured foods will expose the emulsions to a range of ingredients including acids and alkalis. In order to assess whether these changes could cause emulsion destabilisation, aqueous phases were adjusted to pH 3, 6, and 9 prior to homogenisation and the stability of the emulsions formed were assessed. Figure 7 shows the volume mean emulsion droplet diameters of the pH adjusted emulsions after one day and four weeks of storage alongside data acquired on an emulsion formed with pure water as the aqueous phase. Altering the pH of the aqueous phase between 3 and 9 did not have a significant effect on the mean emulsion droplet size over the storage period.

## 3. Materials and Methods

### 3.1. Materials

Ground coffee waste produced from a variety of ground coffee products was collected from a local coffee outlet. Sodium azide (Sigma-Aldrich, Dorset, UK) was added as antimicrobial agent to all aqueous emulsion phases to give a final concentration of 0.02% w/w. Double distilled water was used for all samples. The oil phase of the emulsions varied in composition of isopropyl myristate (IPM) and dodecane (Sigma-Aldrich, Dorset, UK) and commercially available sunflower oil (purchased at a local supermarket). Acetyl bromide (Sigma-Aldrich, Dorset, UK), glacial acetic acid (Fisher Scientific, Loughborough, UK), and low sulfonate kraft lignin (Sigma-Aldrich, Dorset, UK) were used to quantify the lignin content of the milled ground coffee waste particles and hydrothermally treated ground coffee waste particles. Hydrochloric acid (SG 1.16, 32%) (HCl) (Fisher Scientific, Loughborough, UK) and sodium hydroxide pellets (NaOH) (Fisher Scientific, Loughborough, UK) were used to adjust the pH of the aqueous phase to pH 3, 6, and 9. All of these materials were used as received.

### 3.2. Prepartation of Ground Coffee Waste Particle Preparation

Pickering particles were prepared from this material after drying in a convection oven at 40 °C for 48 h to a moisture content of 7.3% ± 0.2% and milling by dry grinding in a planetary ball mill (PULVERISETTE 5 classic line, Fritsch GmbH, Oberstein, Germany) for particle size reduction. The milling conditions were 10 zirconium oxide (ZrO_2_) balls 15 mm in diameter and 70 g of ground coffee waste particles in a 500 mL ZrO_2_ grinding bowl at a main disc speed setting of 200 rpm for 12 h. The milling programme consisted of 5 min intervals of milling and no milling to minimize heat production. Hydrothermally treated milled ground coffee waste particles were prepared by hydrothermally treating the milled waste coffee particles using a protocol adapted from literature [24]. 4 g of sample was mixed with 40 ml of water and sealed into stainless steel tubular reactors (17 cm long and 3 cm inner diameter). Loaded reactors were held at selected temperatures between 150 °C and 275 °C for 1 h. At the conclusion of the treatment, the tubes were cooled by submerging in cold water for 5 min. Solids were retained by filtration (Whatman Grade 1, Kent, UK) and dried in a convection oven at 40 °C for 48 h to a final moisture content of 5.5% ± 0.1%.

### 3.3. Emulsion Processing

All emulsions regardless of the components were prepared as follows. Emulsions were produced on a 50 g scale, containing 46% oil, 46% double distilled water, and 8% particles, based on preliminary experiments (data not presented) where it was found that emulsions containing less than 8% particles were unstable to coalescence. Particles were added as a powder on top of the water phase (the densest liquid phase) followed by the oil phase, in accordance with the powdered particle method [42] as this removes any effect of the initial location of the particles on their wettability which could influence the type of emulsion formed (o/w or w/o). The mixture was emulsified using a high shear overhead mixer (L5M Series fitted with emulsor screen, Silverson, Chesham, UK) operating at 9000 rpm for 2 min. The emulsion type was confirmed by observing whether a drop of the emulsion dispersed in pure oil or in pure water, with a w/o emulsion dispersing in oil and an o/w emulsion dispersing in water [42].

### 3.4. Characterisation of Ground Coffee Waste Pickering Particles and Pickering Emulsions

#### 3.4.1. Lignin Quantification

The milled ground coffee waste and hydrothermally treated (250 °C, 1 h) coffee waste particles’ lignin content was quantified by initially extracting the lignin using acetyl bromide followed by measurement of absorbance (UV-Vis Spectrophotometer, Varian Cary 50, Agilent Technologies, Santa Clara, CA, USA) at 280 nm [24]. Briefly, 100 mg of sample material was dissolved in 4 mL of solvent (25% acetyl bromide, 75% glacial acetic acid) followed by incubation at 50 °C for 2 h. Quantification was performed by calibration using the low sulphonate kraft lignin as a reference material.

#### 3.4.2. Microstructure Imaging

The surface structure of the untreated, milled and hydrothermally treated ground coffee waste particles was investigated using SEM (JSM 6060LV, JEOL, Tokyo, Japan). The particles were placed on SEM stubs with carbotape followed by drying under vacuum before being coated in gold using gold splutter (Leica SCD 0005, Leica Microsystems, Milton Keynes, UK). The samples were then transferred to the SEM stage for imaging.

The microstructure of emulsions was visualised utilizing bright field microscopy (EVOS f1, AMG, Washington, DC, USA) with the aim to support particle sizing data for water continuous emulsions. In the case of oil-continuous emulsions, bright field microscopy was the only method applied to get an insight into droplet size.

#### 3.4.3. Particle Sizing

Size distributions for the aqueous suspensions of waste coffee particles and oil-in-water emulsions were acquired with a low angle laser diffraction particle size analyser (LS 13 320, Beckman Coulter, High Wycombe, UK) fitted with a dispersion cell filled with water (Universal liquid module, LS13 320, Beckman Coulter, High Wycombe, UK). Three independent replicates of each sample were taken. Data was analysed using the Fraunhofer diffraction model using the instrument’s software. Oil-continuous emulsions were not analysed in this equipment due to the reported shear sensitivity of these emulsions and potential droplet size reduction due to the mixing and pumping processes in the dispersion cell.

#### 3.4.4. Particle Hydrophobicity

Particle hydrophobicity was evaluated through an emulsion assay where the lipophilic emulsion phase is varied in polarity and water is the hydrophilic emulsion phase. Following the emulsion preparation method described in 3.3 removing volume fraction of either emulsion phase and initial location of particle addition as impacting factors, the type of emulsion formed provides an indication of particle hydrophobicity as the particles will transfer into the continuous emulsion phase during emulsification, with hydrophilic particles stabilising an o/w emulsion and hydrophobic particles stabilising a w/o emulsion. The polarity of the oil phase was altered to contain varying quantities (0%, 25%, 50%, 75%, and 100%) of IPM, a polar oil, and dodecane, a non-polar oil, as it has been shown in emulsions stabilised with silica particles of intermediate hydrophobicity the nature of the oil can affect the final type of the emulsion formed. Binks et al. [43] found that a polar oil e.g., IPM, interacts more strongly with water; the strength of the interaction can be quantified in terms of the work of adhesion and is calculated from the interfacial tension of the system. As the adhesion between the phases increases, due to an increase in polarity, the water fraction at which phase inversion also increases, therefore for the most polar oils the work of adhesion is too high so no phase inversion occurs allowing only w/o emulsions to be formed whereas non-polar oils preferentially form o/w emulsions. However, as we will show, the hydrophobicity of the Pickering particles can overrule this affect.

#### 3.4.5. Process Stability of o/w Emulsions

To evaluate whether o/w emulsions stabilised by milled ground coffee waste Pickering particles could be successfully incorporated into manufactured food products, sunflower oil–in-water emulsions stabilised with milled ground coffee waste particles were subjected to shear and heat as well as acidic and alkaline conditions as relevant to process steps such as mixing, pumping, pasteurisation, and pH adjustment to achieve desired product textures or to contribute to microbial stability of the product.

The shear stability of the emulsions was evaluated at 20 °C using a rotational rheometer (MCR301, Anton Paar, Graz, Austria) fitted with a concentric cylinder geometry (bob diameter: 27 mm, bob length: 40 mm, cup diameter: 29 mm) applying the following shear ramp protocol. The shear rate was increased from 0.1 to 1000 s^−1^ in 5 min followed by a shear rate decrease from 1000 to 0.1 s^−1^ in 5 min. The shear ramp was repeated three times with the viscosity recorded and plotted against shear rate for all shear ramps.

The influence of temperature on emulsion stability was examined by placing the emulsions in individual glass vials followed by incubation in a water bath at 80 °C for 10 min. The pH of the aqueous phases of the emulsions were adjusted to pH 3, 6, and 9 by adding either HCl or NaOH prior to emulsification. The stability of emulsions subjected to high temperature or different pH environments was evaluated by assessing changes to droplet size and microstructure of the emulsions. Due to the presence of particle aggregates and potentially individual large particles, the particle size data were manipulated to remove any data at sizes greater than 400 µm, therefore only changes to the emulsion droplet size could be assessed.

### 3.5. Statistical Analysis

Mean size and standard deviation are reported based on three independent samples. The particle size data was significantly analysed using an ANOVA and Tukey’s statistical test with the level of significance set at *p* = 0.05 for both statistical tests.

## 4. Conclusions

Waste coffee particles (unmilled and milled) and hydrothermally treated waste coffee particles can act as Pickering particles for both o/w and w/o emulsions. The Pickering stabilisation is a result of the presence and, for w/o application the relocation of the known emulsifying agent, lignin. Pickering particle hydrophobicity assessment confirmed that the relocation of the lignin to the particle surface had increased the hydrophobicity of the particles compared to the hydrophilic untreated particles. However, emulsions stabilised with the hydrothermally treated waste coffee Pickering particles had large droplets which would not be suitable for incorporation into food products, necessitating further research into process optimisation for this application.

On the other hand, the use of milled waste coffee Pickering particles to stabilise o/w emulsions of typical food formulation produced emulsion droplets of desirable size with no change in microstructure seen over a period of 12 weeks of storage. Investigations also showed that the o/w emulsions to be stable to shearing up to 1000 s^−1^ and heating to 80 °C, conditions typical of food product manufacture. Finally, altering the pH of the aqueous phase to between pH 3 and pH 9 was not found not to affect the stability of the emulsions with no change in droplet size seen for a period of four weeks.

Overall, this study has demonstrated that lignin rich food waste can be functionalised as a food ingredient with emulsifying property for both oil and water based foods. While application in water based foods, i.e., for o/w emulsions, appears to be more readily possible and is thus potentially closer to application, further research is required to develop commercially relevant particles for the application in lipid continuous foods or for the stabilisation of the encapsulated water phase in duplex (w/o/w) food emulsions. In addition, the natural abundance of lignin in plant based materials and plant based food waste begs to extend this application to materials other than coffee. Due to the complex and variable structure of lignin, the use of different sources could enable the creation of particles with a range of functionalities and applications.

## Figures and Tables

**Table 1:** Volume-weighted characteristic particle sizes of coffee particles examined for Pickering properties. All particles were dried prior to particle size analysis.

Sample	*d*_4,3_ (μm)	*d*_10,3_ (μm)
Unmilled	341.11 ± 21.67	35.91 ± 5.65
Milled	117.12 ± 16.15	21.90 ± 2.55
Milled and Hydrothermally Treated	144.25 ± 6.14	19.24 ± 0.20
